# Supplementary figures and images for: An electrodiffusive network model with multicompartmental neurons and synaptic connections
Source: PLoS Comput Biol. 2024 Nov 12;20(11):e1012114. doi: 10.1371/journal.pcbi.1012114 (PMC11584141; doi:10.1371/journal.pcbi.1012114)

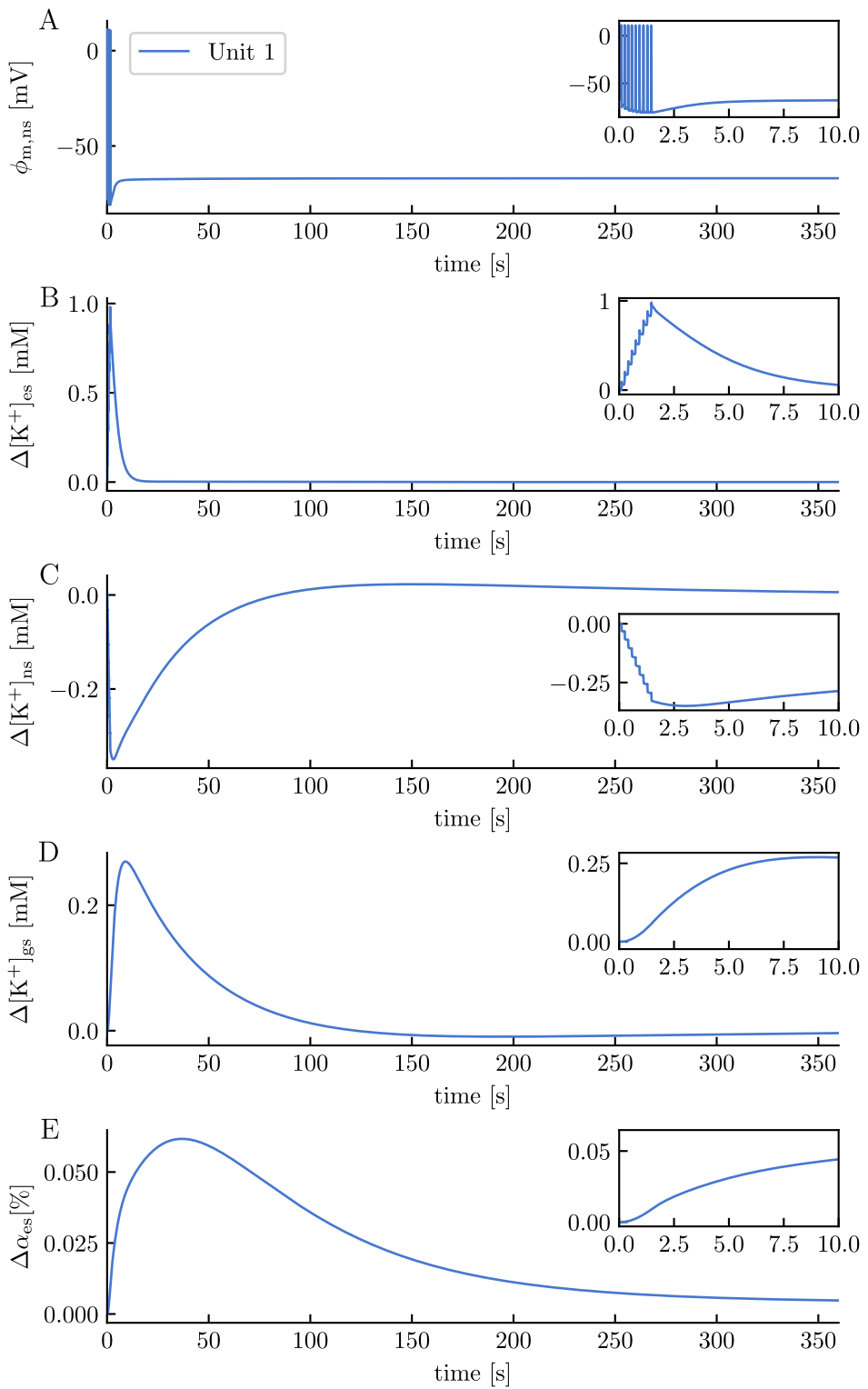

Supplement: S1 Fig — Temporal evolution of the neuronal membrane potential (A) and change in ECS K+ concentration (B), neuronal K+ concentration (C), glial K+ concentration (D), and ECS volume fraction (E) in the soma layer of unit 1. The figure is based on the simulation presented in Fig 2, except all synaptic currents were turned off at t = 1.5 s. (PDF) [file pcbi.1012114.s001.pdf]

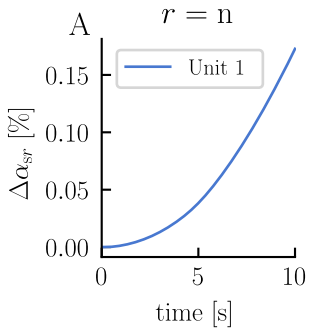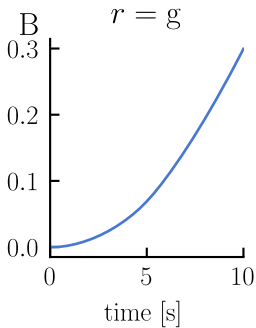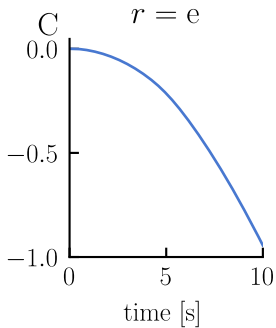

Supplement: S2 Fig — Temporal evolution of the neuronal (A), glial (B), and extracellular (C) volume fractions of the somatic layer in unit 1, given as change from baseline values. The figure is based on the simulation presented in Fig 7. (PDF) [file pcbi.1012114.s002.pdf]
