# Supplementary material for: An electrodiffusive network model with multicompartmental neurons and synaptic connections
Source: PLoS Comput Biol. 2024 Nov 12;20(11):e1012114. doi: 10.1371/journal.pcbi.1012114 (PMC11584141; doi:10.1371/journal.pcbi.1012114)
Supplement: Attachment — Submitted filename: reponse_to_reviewers.pdf [file pcbi.1012114.s003.pdf]

Dear Editor,

We hereby resubmit our manuscript "An electrodiffusive network model with multicompartmental neurons and synaptic connections" by Marte J. Sætra and Yoichiro Mori for publication in PLoS Computational Biology.

We thank the reviewers for their feedback and constructive comments. Our detailed response follows below. The reviewers' comments are included in blue, while our responses follow in black text. Also attached is a version of the manuscript indicating the changes made from the previous version.

Reviewer #1: The paper presents an intriguing extension of the author's prior work, utilizing the Kirchhoff-Nernst-Planck framework to model ion electrodiffusion within a two-compartment neuron, accompanied by glial cells and the extracellular space. They augment this by incorporating synaptic coupling in a classic ring model formation and explore pathological conditions, demonstrating how excessive stimulation can induce depolarization block, a phenomenon they then demonstrate is ameliorated by sufficient astrocytic clearance of extracellular potassium. I recommend the paper for publication, but have a few queries and suggestions for improvement.

Comment: We thank the reviewer for this prompt and positive evaluation of our manuscript.

Reviewer #1: Figure Presentation: It is quite difficult to follow the individual traces. As the plot appears twice, could you show/highlight one example in either Fig2B or Fig3A.

Comment: Thanks for pointing this out. We agree.

Action: We have highlighted the first trace in Fig 3A.

Reviewer #1: Resting Membrane Potential (RMP) Dynamics: It seems that the RMP decreases between each action potential in Fig 2B. Given the expected increase in the Nernst potential for K^+ (with a relatively larger increase in extracellular concentration than the reduction in neuronal concentration), is the change in RMP primarily driven by Na^+ dynamics?

Comment: This is a very good question. While it is reasonable to believe that the effect is driven by the sodium dynamics, in this instance, it is primarily caused by the (Ca-sensitive) afterhyperpolarization current. If we deactivate the AHP channels, the observed change in RMP is heavily reduced, as shown in the figure below.

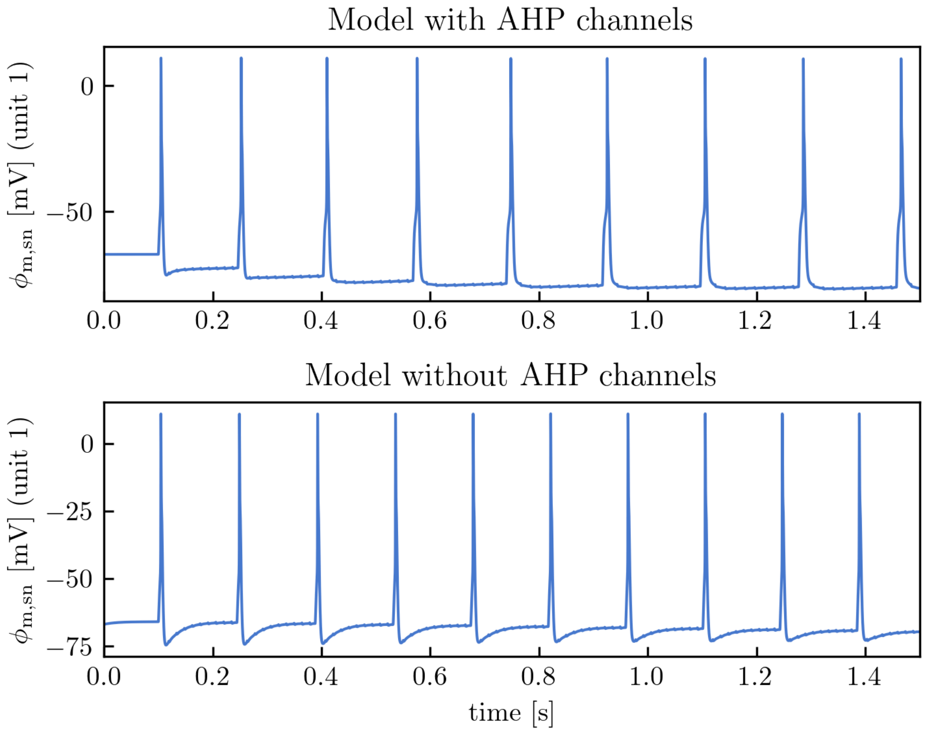

Reviewer #1: Extracellular Volume Fraction Changes: While the change in volume is minor, increased neuronal activity typically results in reduced extracellular volume. Have you observed a reduction in ECS volume in pathological simulations (Fig 7 & Fig 8)? Additionally, what drives the increase in extracellular volume fraction in Fig 4? If Na^+ dynamics contribute, consider including them in a figure or discussing them in the text.

Comment: Thanks for this observant comment. We have indeed observed a reduction (~1%) in ECS volume in pathological simulations and have examined what drives the volume changes in the two scenarios. For the physiological case (Fig 2-4), the volume changes are driven by small changes in osmolarities (<1 mM), dominated by potassium. For the pathological case (Fig 7-8), the changes in osmolarities are of the order of 1-10 mM and dominated by sodium. Since the change in Fig 4H is so minor, we think that it may not be meaningful to delve into the specifics of what drives this change in the manuscript. However, we have opted to slightly rephrase the paragraph to underscore how minor this change is and have included a figure in the supplementary material illustrating the volume changes for the pathological case.

Action: We have added a supplementary figure (S2 Fig) showing volume changes in pathological simulations and expanded the paragraph on cellular volume changes.

Page 6, line 178: "Owing to the relatively brief duration of the simulation and the low firing rate within the current configuration, we detect only a slight change in the ECS volume, approximately 0.01% increase. However, in scenarios with considerably elevated firing rates, our

model predicts shrinkage of the ECS, consistent with empirical observations (S2 Fig) [Dietzel et al. 1980, Ransom et al. 1985]."

S2 Fig. Volume dynamics in pathological conditions. Temporal evolution of the neuronal (A), glial (B), and extracellular (C) volume fractions of the somatic layer in unit 1, given as change from baseline values. The figure is based on the simulation presented in Fig 7.

Reviewer #1: Homeostatic Recovery; Line 161 suggests an expected return to baseline values due to homeostatic activity. Could you clarify if there is any recovery over the short period between spikes, such as in the somatic neuronal K^+ concentration (Fig 4E)? Have you experimented with halting spiking to observe if it returns to baseline or enters another stable state?

Comment: There is indeed some recovery between each spike since the pump is always active, but it is slow. If we halt spiking, the system returns to baseline.

Action: Inspired by your comment, we thought it would be nice to actually illustrate homeostatic recovery of the system. We have slightly modified the paragraph in question and added a supplementary figure.

Page 6, line 168: "... the intraneuronal K^+ concentrations recover more slowly than in the ECS and stay close-to constant between each AP. However, over longer periods of inactivity, the intracellular ion concentrations return to baseline values together with the rest of the system (S1 Fig)."

S1 Fig. Homeostatic recovery of the system. Temporal evolution of the neuronal membrane potential (**A**) and change in ECS K^+ concentration (**B**), neuronal K^+ concentration (**C**), glial K^+ concentration (**D**), and ECS volume fraction (**E**) in the soma layer of unit 1. The figure is based on the simulation presented in Fig 2, except all synaptic currents were turned off at t = 1.5 s.

Reviewer #1: Calcium Flux: Considering the low concentration of calcium involved, displaying the calcium flux on a different scale in Fig 6A,B might enhance clarity.

Action: Thanks for the suggestion. We have updated the figure.

Reviewer #1: Astrocyte K^+ Buffering: Regarding the representation of inactive regions in the model, particularly when u=2, could the use of a 1D simulation exaggerate concentration changes due to volume of available tissue increase with the cube of the distance from the active region? While this may not impact the conclusion about potassium buffering preventing SD, it might be worth noting in the text for clarity.

Comment: Thanks for pointing this out. Indeed, the effect of a 1D setup on ion concentrations (and electrical potentials) should be mentioned somewhere.

Action: We have added the following statement to the Discussion section:

Page 11, line 367: "We acknowledge that the choice to position the units in a 1D configuration can lead to somewhat high amplitudes in the extracellular AP signatures and exaggerated ion concentration changes. This outcome is a consequence of the increased total resistance (limited degrees of freedom) for extracellular currents and a reduction in the volume of tissue available."

Reviewer #1: ECS Size: The diagram in figure 10A suggests that the ECS size remains constant regardless of glial presence, but Equation (2) implies it should be larger without glia. Please clarify this in the caption.

Comment: Thanks for pointing this out. The reviewer is absolutely correct regarding Eq 2. However, we constructed the no-glia model by disabling the membrane mechanisms (cf. line 292), thereby preserving the volume fractions.

Action: We have added short descriptions of the models to the figure caption to avoid any potential confusion:

Fig 10: "...for the no-glia (i.e., no glial membrane mechanisms), weak-glia (i.e., weak gap junctions), and strong-glia (i.e., strong gap junctions) model…"

Reviewer #1: Simulation details: You note in the discussion that a 2D or 3D model would be computationally demanding, I am curious how computational intensive these 1D simulations were. Could you add how long the simulations took to run in Table 4, or comment on it in the text.

Comment: Good idea - since we argue that compartmentalized models are more computationally manageable than geometrically explicit models it makes sense to report the simulation runtimes.

Action 1: We have added simulation runtimes to Table 4 and updated the section on simulation details accordingly. The section now says:

Page 20, line 568: "A summary of the different simulation protocols used in this paper is given in Table 4, including approximate simulation runtimes. Note that we utilized the Numba library (Lam et al. 2015) to speed up computations, and the reported runtimes do not include ahead-of-time compilation times. Timings were conducted on a Lenovo ThinkPad X1 Carbon Gen 8 with an Intel Core i7-10510U CPU at 1.80 GHz and Ubuntu 22.04.4 LTS."

Action 2: We have slightly rephrased the discussion on computational demand. It now say:

Page 12, line 421: "Expanding the model's dimensionality would not present a substantial mathematical challenge; however, large-scale network simulations would necessitate careful

consideration during model implementation to keep computational demands within manageable limits. This is particularly crucial when calculating the synaptic conductances (Srinivasan and Chiel 1993)."

Reviewer #2: In this manuscript, the authors present a network level model of electrodiffusion suggesting that this model bypasses the need to consider neuronal geometry but can still predict ion concentrations. They develop a comprehensive multicompartment model and test different scenarios. However, how the simulation case studies or the motivation is biologically relevant is unclear as written. I have the following major concerns or questions:

1) Introduction: the motivation is given by the fact that even though many models exist, they cannot be used to study the spread of depression. But then I didn't find any evidence of what happens to ionic concentrations in depression or that this model has the potential to shed light on depression. The introduction teases at different important scenarios but doesn't clearly make the case for what scenarios (with experimental measurements) motivate their model. They also talk about computationally expensive spatial models but how expensive is expensive?

Comment: We believe this comment stems from the reviewer's unfamiliarity with the phenomenon of spreading depression, which is a wave of hyperactivity followed by neuronal silence, or "depression." This phenomenon is associated with substantial changes in both ion concentrations and extracellular potentials (cf. ref. [6,7,8,9]). We are not suggesting that no models can be used to study spreading depression. On the contrary, the domain-type models we discuss in the introduction are good candidates. However, due to their homogenized nature, these models do not allow for tracking the activity of individual cells (cf. line 47). On the other end of the spectrum are (compartmentalized) single-cell models, which naturally cannot be used to study how spreading depression propagates through tissue (cf. line 36). Our model offers a bridge between the single-cell and domain type frameworks, and while the focus in this paper is not on spreading depression, our model could potentially be used to study it.

The electrodiffusive network model does not bypass the need to consider neuronal geometry. Indeed, there are cases where examining explicit geometries is highly relevant. However, using explicit geometries to simulate networks of cells is prohibitively expensive. By compartmentalizing the system, we make such simulations feasible.

Action 1: We have added a short description of spreading depression to the introduction:

Page 2, line 14: "... spreading depression (a slow wave of hyperactivity followed by neuronal silence associated with, i.a., migraine aura (Leão 1944, Dreier 2011))."

Action 2: We have slightly modified the paragraph on geometrically explicit models. It now says:

Page 3, line 38: “These types of models are superior when it comes to geometrical detail and to uncover relationships between shape and function. The models can in theory be expanded to include an arbitrary number of cells, but the computational cost of simulating hundreds of neurons is prohibitively expensive.”

Reviewer #2: Typos: line 63 and line 35.

Action: Fixed.

Reviewer #2: 2) Figure 1: I had a very hard time understanding the compartments in Figure 1. I understand soma and dendrites for neurons. What does each unit represent? That every neuron is paired with a glial cell? Are there no units with just neurons and ECS?

Comment: All units come with one neuronal domain paired with a glial domain and extracellular space. These units may represent multiple neurons/glia grouped together or a single neuron/glia, depending on parametric values. We agree that this is not a faithful representation of neural histology. However, for purposes of studying spreading depression and other bulk neuronal phenomena, this picture still provides useful information. Moreover, electrodiffusion simulations performed in our paper are much more computationally intensive than typical electrophysiology simulations.

Action: We have added the following statement to enhance clarity:

Page 3, line 81: “Each unit can be interpreted in one of two ways, depending on parametric values (Sætra et al. 2021). It could represent a single neuron and the average piece of ECS and glial ion uptake available to that neuron. Alternatively, it could represent an average piece of tissue, comprising multiple neurons grouped together along with their shared ECS and glial ion uptake. In this paper, we treat each neuronal domain as representing a single neuron and its characteristics within the somatic and dendritic layers”.

Reviewer #2: 3) Parameter choices: The authors mention that they tune parameters manually (line 128) to obtain certain outcomes but I didn't see any mention of parametric sensitivity analysis? 4) The model has many free parameters, how do we know that any of the effects shown are robust?

Comment: We only tuned the coupling strength between layers to preserve the characteristic action potential shape of the Pinsky-Rinzel model. This parameter had to be set/tuned when we transitioned from explicit areas and volumes (Sætra et al. 2021) to volume fractions and area-to-volume fractions (this paper). All other parameters were adopted from our previous publication (Sætra et al. 2021). We think it is relevant to demonstrate that the characteristic action potential shape can be maintained when the cell model is re-implemented and transitioned into a network setting. Beyond this, we do not expect this feature to influence the remaining

content of our paper. The primary objective of our paper is not to present quantitative results, but to introduce a framework for network modeling that incorporates ion concentration dynamics and includes all relevant physics. Furthermore, we aim to gain a qualitative understanding of the potential behaviors the model can exhibit based on the inherent physics, which is present in the model regardless of what parameters we choose. We certainly recognize that sensitivity analysis is a crucial part of model development and have recently published a paper on the topic (Signorelli 2024, PloS ONE). However, we consider such an analysis to be outside the scope of this paper.

Reviewer #2: 5) Line 139: What is the functional consequence of drop in glial membrane voltage at t=1.5 s?

Comment: The slow increase in glial membrane voltage is a consequence of ion concentration shifts and affects the glial membrane currents.

Reviewer #2: 6) Results: This comment summarizes the overall concern I have with the paper. In figure 3C, the authors zoom in and show an extremely small difference and suggest that it is important. I have a very hard time understanding why differences that are practically negligible interesting and important. If one were to add noise to this system, would any of these outcomes hold? Later, there are various simulations of reduced models and comparison models. I am left wondering -- what is the point? The differences are so small that I don't know how to interpret any of the effects.

Comment: We are not claiming that the small difference we observe in Fig 3C is of substantial importance. Indeed, as we note in the introduction, "the extracellular potential is typically so small that one can neglect ephaptic effects on neurons without severe loss in accuracy" (cf. line 9). However, there are several reasons why we include extracellular potentials in our model.

Firstly, as we discuss in the introduction, there are scenarios where extracellular potentials can reach several tens of millivolts in magnitude. Such settings cannot be studied with conventional models where the extracellular potential is assumed to be spatially uniform and grounded.

Secondly, our aim was to construct a network model with ion concentration dynamics that encompasses all relevant physics and maintains biophysical consistency in the relationship between ion concentrations, electrical potentials, and charge. Ion concentrations are often studied by considering diffusion alone, but as we have discussed in previous work (Sætra et al. 2020) and touch upon in Fig 11, it is not a given that contributions from electric field effects are negligible, even when extracellular potentials are not particularly large. Our model can also be used to study the effects of diffusive currents on extracellular potentials, which is also a topic we have discussed in previous work for single cells (Sætra et al. 2020, Sætra et al. 2021). In the context of performing forward modeling of extracellular potentials, such as local field potentials,

ECoG, and EEG, the standard approach is to first calculate the neurodynamics assuming that the extracellular potential is zero. Then, this neurodynamics is used to predict a *nonzero* extracellular potential. In this approach, it is assumed that the contribution from diffusive currents is negligible. Our model offers both a more biophysically accurate method for predicting extracellular potentials and a tool for assessing the potential loss in accuracy when assuming negligible diffusive currents.

Lastly, we think that our model can also serve an educational purpose. By encompassing all relevant physics, it can contribute to a better understanding and intuition of electrophysiology.

The purpose of the various examples that follow is primarily to demonstrate the different properties of the model that result from including both ion concentration dynamics and extracellular potentials. We disagree with the claim that these effects are vanishingly small. Fig 5 shows a substantial difference in firing rate, Fig 7 shows that neurons can fire spontaneously, and Fig 9 demonstrates that strong glial coupling can prevent depolarization blocks. While these differences are primarily driven by changes in ion concentrations, it is impossible to completely decouple ionic and electrical ephaptic effects since ion concentrations and electrical potentials influence each other.

Action: We have added the following paragraph to better communicate the point of offering an electrodiffusive network model:

Page 12, line 402: "We envision that the electrodiffusive network model will benefit the neuroscience community in three main ways. Firstly, by relaxing some common model assumptions, the model can be used to evaluate the validity of these assumptions under various conditions. For instance, we can determine when it is necessary to consider the effect of electric drift on ion concentrations or when to take into account the impact of diffusive currents on extracellular potentials. This is a topic we have also discussed in previous work for single cells (Sætra et al. 2020, Sætra et al. 2021). Secondly, we think that our model is foundational in its incorporation of all relevant physics and can thus lead to a deeper understanding and intuition of electrophysiology. Thirdly, the electrodiffusive network model can be useful for exploring a variety of scenarios influenced by ion concentration dynamics."

Reviewer #2: 7) How is the reader to understand the different network configurations? I just really had a hard time building confidence in the biological importance of these findings.

Comment: We are not trying to draw any conclusions that are specific to neuronal network topology. As the reviewer suggests, our model is too coarse to address such issues. What is at issue here is the effect of spatiotemporal changes in ionic concentration and how they are affected, for example, by the presence/absence of glia. For these issues, we believe that our model provides sufficient insight. We do believe, as the reviewer suggests, that network topology

is important. This is a topic for future exploration and is far beyond the scope of the current manuscript.

Reviewer #3: The authors present the results from an electro-diffusive neuronal tissue model. The model incorporates compartments for neuronal, glial and extracellular spaces that they couple in multiple ways. The complexity of the neuronal models is moderate and the results are consistent with previous work. The incorporation of electrodiffusive dynamics is an important step forward for neuronal network models, but I feel that the manuscript needs to address a few small changes. Aside from potential typos I may have missed, the model looks reasonable under the time scale/space scales involved here.

Comment: We appreciate the positive evaluation of our manuscript.

Reviewer #3: The assumptions in the physical model appear sound, but it would be good to provide more physiological motivation for the different models, i.e. no/weak/strong glial coupling. For instance what is the physiological interpretation of a diffusion length of 1 mm?

Comment: This is a very understandable input. In this paper, our goal is to introduce a framework for network modeling with ion concentration dynamics that includes all relevant physics and maintains biophysical consistency between ion concentrations, electrical potentials, and charge - without focusing on specific phenomena at this time. The purpose of the various examples is rather to show the model's properties and demonstrate how the model differentiates from conventional models. Our primary goal is to build an understanding of the model and to develop an intuition around how electrical potentials, ion concentrations, and volume dynamics are tightly coupled. We also want to give the reader an idea of the settings in which this model could be relevant. While we have attempted to describe how the various setups should be interpreted and think they hold biological relevance, we also do not want to overstate their physiological motivation.

Regarding the model with no/weak/strong glial coupling, we wanted to make some qualitative predictions of the importance of including glia in the model. A diffusion length of 1mm is not meant to be interpreted literally, but rather, it indicates that the coupling between the two units is "weak" or "not efficient". This could imply that they are "far apart", but we could also have adjusted the diffusion in other ways, such as changing the tortuosity, or as we have done for the intracellular space, multiplied the flux by a number that we envision represents the strength/number of gap junctions.

Reviewer #3: The model is capable of producing ephaptic ionic and field effects. However it is not clear that the ionic part of this coupling is any different than the ionic coupling incorporated in many other network models.

Comment: The reviewer is correct in the sense that ion concentrations influence cell activity in our model in the same way as they do in other network models with ion concentration dynamics (i.e., via changes in the ionic reversal potentials). What sets our model apart from others is the *coupling* between ephaptic ionic and field effects, where the electric fields influence the ionic transport and the ion concentrations, in turn, affect the electric fields. In this way, we take into account all relevant physics when calculating ion concentrations, thus achieving biophysical consistency and more accurate predictions of ion concentrations. While diffusion often dominates ion transport over electric drift, this is not always the case, a point we discuss in Fig 11 and in previous work (Sætra et al. 2020).

Reviewer #3: The general outline of the KNP framework should be concisely reviewed/described in this paper. It is important to the understanding of the physics/physiology of the model.

Action: We have added additional statements to the Methods section to highlight the KNP framework's underlying physics/assumptions. Please see marked changes on page 12 and 15 in the revised manuscript.

Reviewer #3: Figure 7. Partially claims that the ephaptic coupling gives rise to the multiple action potentials, however they are gradually depolarizing up to this point and seem to just be reaching their thresholds within close temporal proximity.

Comment: The gradual depolarization of the neurons is indeed a result of the (ionic) ephaptic coupling. Without this effect, the neurons would maintain their resting membrane potential throughout the simulation and never reach their firing thresholds. The membrane potentials could also increase if the system was not sufficiently calibrated or had a resting membrane potential above the firing threshold. Therefore, we have added a sentence emphasizing that the system starts in a steady resting state.

Action: We have added the following statement:

Page 8, line 241: "Before stimulus onset, all neurons maintain a resting membrane potential of -67 mV."

Reviewer #3: The authors don't show the results for their model including diffusion, but lacking drift. The advancement of the model over previous models is the drift, so this comparison would help to elucidate the effects of the ephaptic field effects. In general the effects of the ephaptic field are small, except in the extracellular drift for strong glia, and they are not highlighted by the authors. As the main advancement to the model, the authors could do more to give support for its necessity.

Comment: We agree that it is meaningful to compare the effects of diffusion versus electric drift. However, due to the way our model's framework is constructed, it is technically impossible to turn off the drift component while retaining diffusion. To do this, one would have to utilize a different set of equations in which electric potentials are decoupled from ion concentrations, set to zero in the extracellular space, and computed based on the transmembrane- and intracellular (drift-driven) currents.

What is possible with our framework, for instance, is to compare drift- and diffusion-driven currents, as in Fig 11. Here, we demonstrate that the two components are of the same order of magnitude for the extracellular space in the strong-glia model and that drift dominates glial transport in the weak-glia model. We have also discussed the role of electric drift in previous work (Sætra et al. 2020). Whether drift plays a substantial role in ionic transport is highly context-dependent, and our model gives researchers a tool to predict when it is necessary to incorporate drift into their models and when it can be omitted.

Please see our comment and **action point** to Reviewer 2's sixth comment for further discussion of extracellular potentials and electric drift.

Reviewer #3: Figure 8. Heat maps don't add much to the story.

Comment: We think it is necessary to show how the extracellular potassium concentrations and reversal potentials change across all the units to demonstrate the presence of ionic ephaptic effects.

Reviewer #3: Figure 9. Is there anything to see in the unit 2 voltage? If they don't change, perhaps best to leave them off.

Action: Fixed.

Reviewer #3: Line 518 "This approach guarantees conservation of ions." This should be explained in the text. The reference doesn't claim to conserve, only minimize disruption.

Comment: By "this approach," we are referring to the method of transferring ions across the membrane as opposed to introducing additional ions into the system, but we see that our current explanation is unclear. Thanks for pointing this out.

Action: We have removed the statement to avoid confusion.
